# ﻿*Xenosyangi* sp. nov.: A new twisted-wing parasite species (Strepsiptera, Xenidae) from Gaoligong Mountains, Southwest China

**DOI:** 10.3897/zookeys.1085.76484

**Published:** 2022-02-02

**Authors:** Zhiwei Dong, Xingyue Liu, Chuyang Mao, Jinwu He, Xueyan Li

**Affiliations:** 1 State Key Laboratory of Genetic Resources and Evolution, Kunming Institute of Zoology, Chinese Academy of Sciences, Kunming, Yunnan 650201, China Kunming Institute of Zoology, Chinese Academy of Sciences Kunming China; 2 Department of Entomology, China Agricultural University, Beijing 100193, China China Agricultural University Beijing China; 3 Kunming College of Life Science, University of Chinese Academy of Sciences, Kunming, Yunnan 650204, China University of Chinese Academy of Sciences Kunming China

**Keywords:** Mitogenome, morphology, new species, taxonomy, wasp endoparasite

## Abstract

Here we report a new twisted-wing parasite species of the family Xenidae based on both morphological and molecular evidence. By using nearly complete mitogenomes, we confirmed the twisted-wing parasites on two wasps (*Vespavelutina* and *Vespabicolor*) (China: Yunnan) as the same species, and associated its neotenic females and alate males. Combining the mitogenomic data (*COI*) and morphological traits, this species was identified to be a new species of the genus *Xenos*, namely *Xenosyangi* Dong, Liu & Li, **sp. nov.** Detailed descriptions and illustrations are provided for the new species.

## ﻿Introduction

Strepsiptera are obligate endoparasites of silverfish, cockroaches, mantids, orthopterans, hemipterans, wasps, bees and flies, and they comprise about 630 species in 15 families (Kathirithamby 2018). Among 10 extant families, Xenidae Saunders, 1872 uses wasps as hosts and is the one of the species-rich strepsipteran families with ca 110 described species in four genera (*Paragioxenos* Ogloblin, 1923; *Paraxenos* Saunders, 1872; *Pseudoxenos* Saunders, 1872; *Xenos* Rossius, 1793) (Pohl and Beutel 2008; Cook 2014; Benda et al. 2019). Benda et al. (2019, 2021) comfirmed the paraphyly of *Pseudoxenos* and polyphyly of the genera *Xenos* and *Paraxenos* using molecular data. The genus *Xenos* is one of the twisted-wing insects parasitic on eusocial wasps (Pohl and Beutel 2008; Kathirithamby 2018) and contains 41 species worldwide (Suppl. material 1: Table S1). About two-third (26 species) of *Xenos* species are distributed in the Americas, while the remaining 15 species are distributed in Africa (five species), Africa/Europe (one species) and Asia (nine species) (Buysson 1903; Kifune and Maeta 1985; Yang 1999; McMahon et al. 2009; Cook 2019; Cook et al. 2020; Kathirithamby 2021) (Suppl. material 1: Table S1). Among nine Asian species, five are recorded in China [*Xenosmoutoni* (Buysson, 1903): Yunnan, Anhui, Taiwan; *X.circularis* Kifune & Maeta, 1985, *X.yamaneorum* Kifune & Maeta, 1985 and *X.formosanus* Kifune & Maeta, 1985: Taiwan; *X.dianshuiwengi* Yang, 1999: Fujian], two in Japan [*Xenosvespularum* Kifune & Maeta, 1975 and *Xenosoxyodontes* Nakase & Kato 2013], one in India [*Xenoshebraei* Kinzelbach, 1978] and one in Indonesia [*Xenosprovesparum* Kifune, 1986] (Buysson 1903; Kifune and Maeta 1985; Yang 1999; Cook 2019). *Xenosvesparum* Rossius, 1793, which is the type species of both this genus and all strepsipteran insects (Rossius 1793), is a well-studied species with abundant data on its morphology and biology (Kifune and Maeta 1985; Manfredini et al. 2007; Nakase and Kato 2013; Richter et al. 2017).

**Figure 1.**
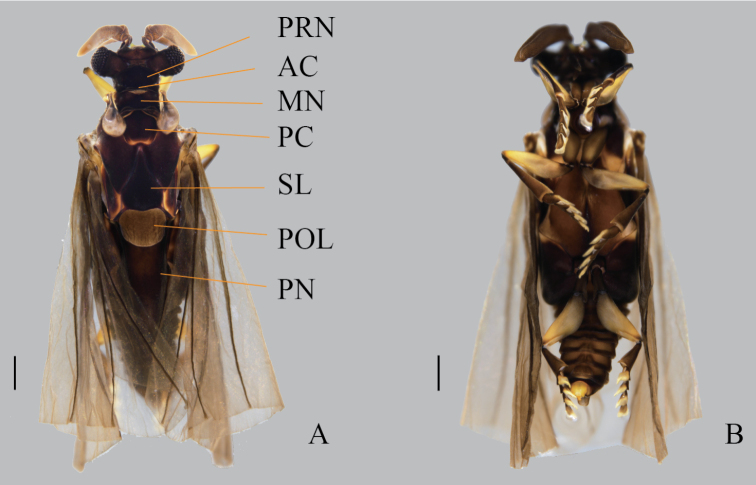
*Xenosyangi* Dong, Liu & Li sp. nov., male adult (holotype) **A** dorsal view (PRN, Pronotum; AC, Acrotergit; MN, Mesonotum; PC, Prescutum; SL, Scutellum; POL, Postlumbium; PN, Postnontum) **B** ventral view. Scale bar: 0.5 mm.

**Figure 2.**
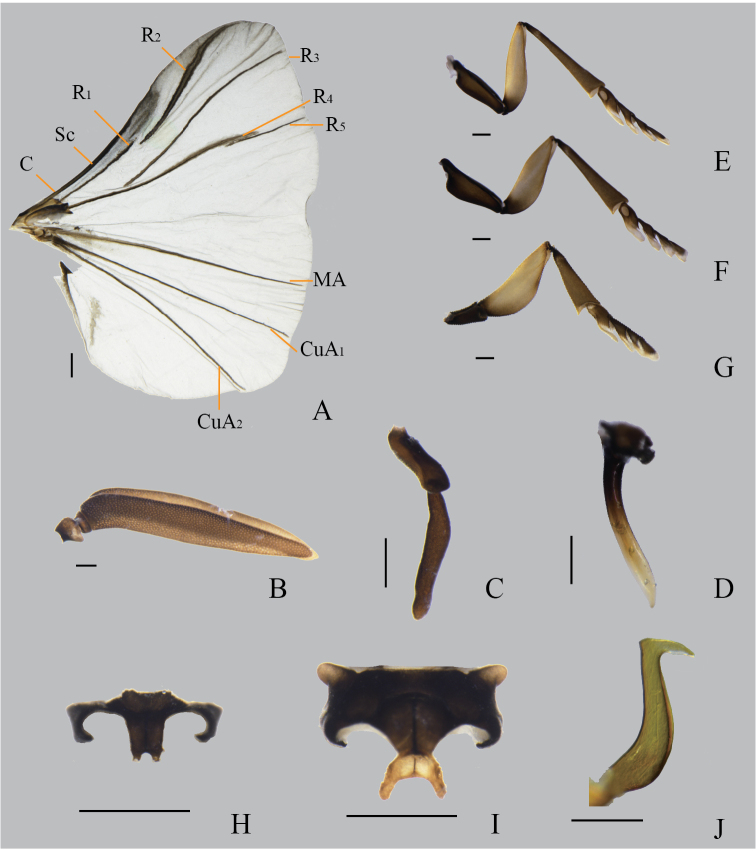
*Xenosyangi* Dong, Liu & Li sp. nov., male adult **A** hind wing **B** right antenna **C** right maxilla and palpus**D** right mandible **E** foreleg (right) **F** midleg (right) **G** hind leg (right) **H** proventrite **I** mesoventrite **J** penis. Scale bars: 0.5 mm. **A, B** dorsal **H, I** ventral **C–J, F** lateral.

**Figure 3.**
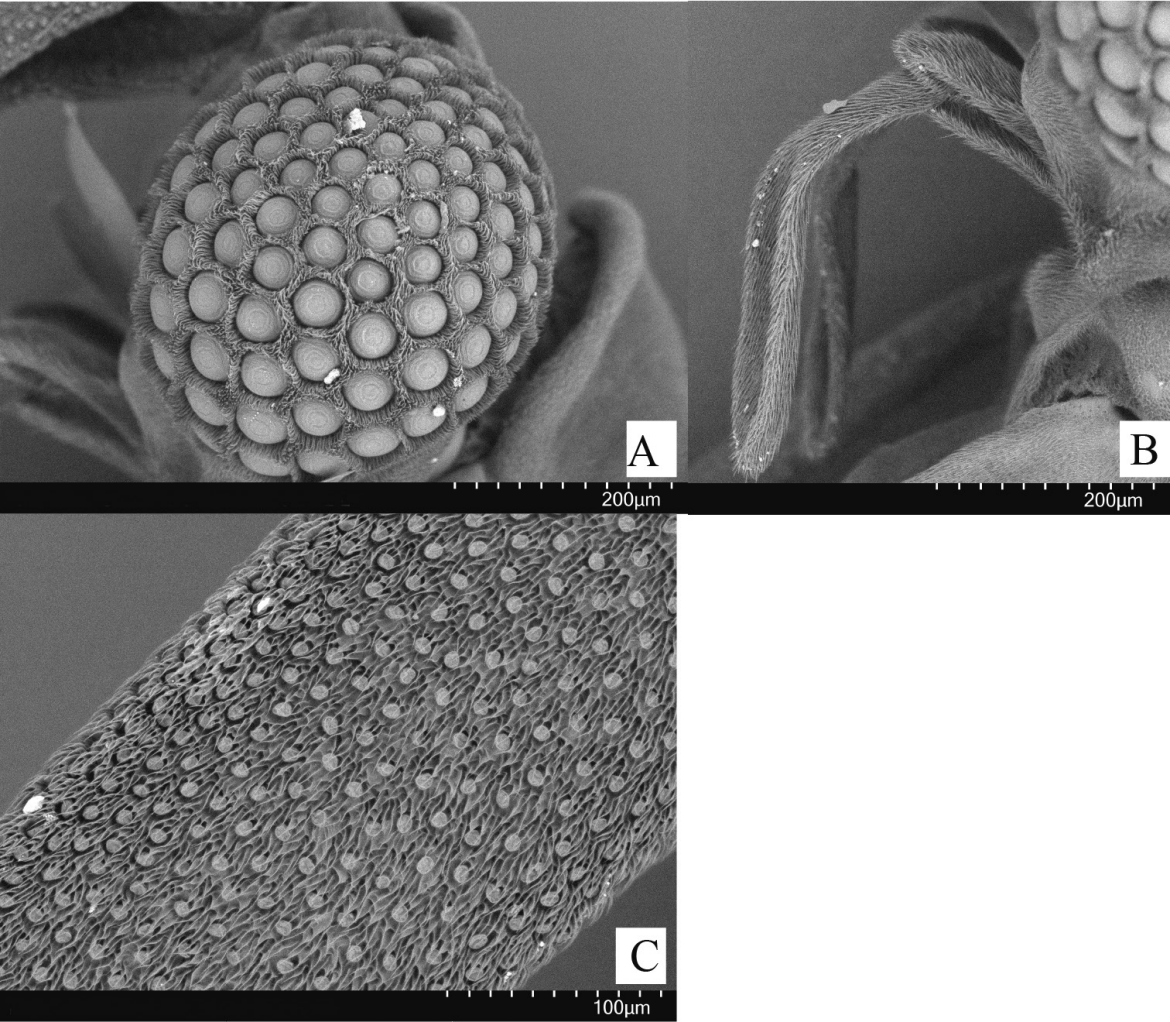
*Xenosyangi* Dong, Liu & Li sp. nov., male adult (SEM micrographs) **A** compound eye (lateral) **B** maxilla and palpus (lateral) **C** fourth antennomer (dorsal).

**Figure 4.**
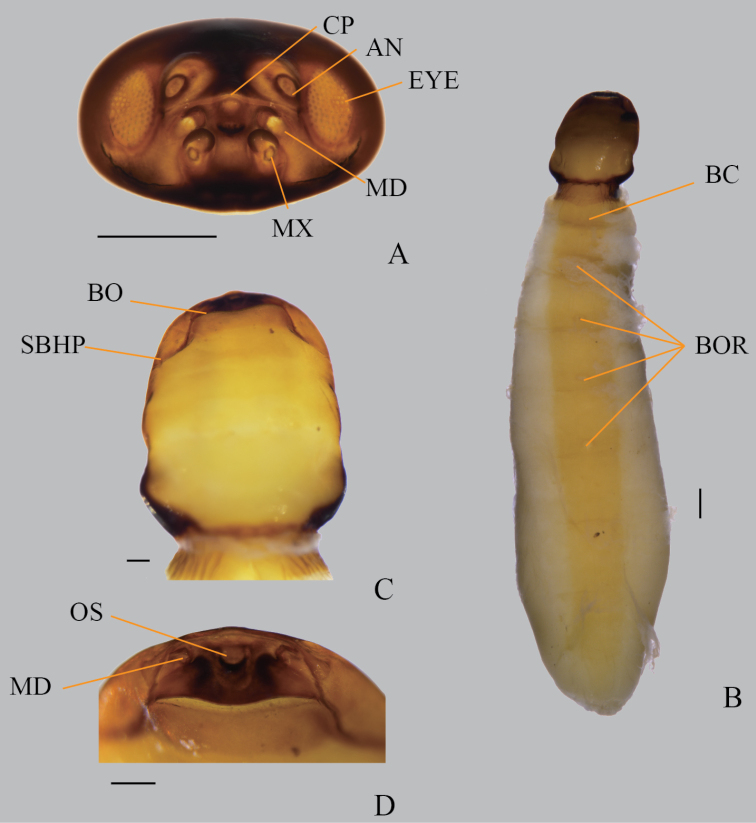
*Xenosyangi* Dong, Liu & Li sp. nov. **A** male cephalotheca frontal view (CP, Clypeus; AN, Antenna; EYE, Eye; MD, Mandible; MX, Maxillae) **B** female ventral view (BC, brood canal; BOR, birth organs) **C, D** female cephalothorax ventral view (BO, birth opening; SBHP, segmental border between head and prothorax; OS, mouth opening; MD, mandible). Scale bar: 0.5 mm.

**Figure 5.**
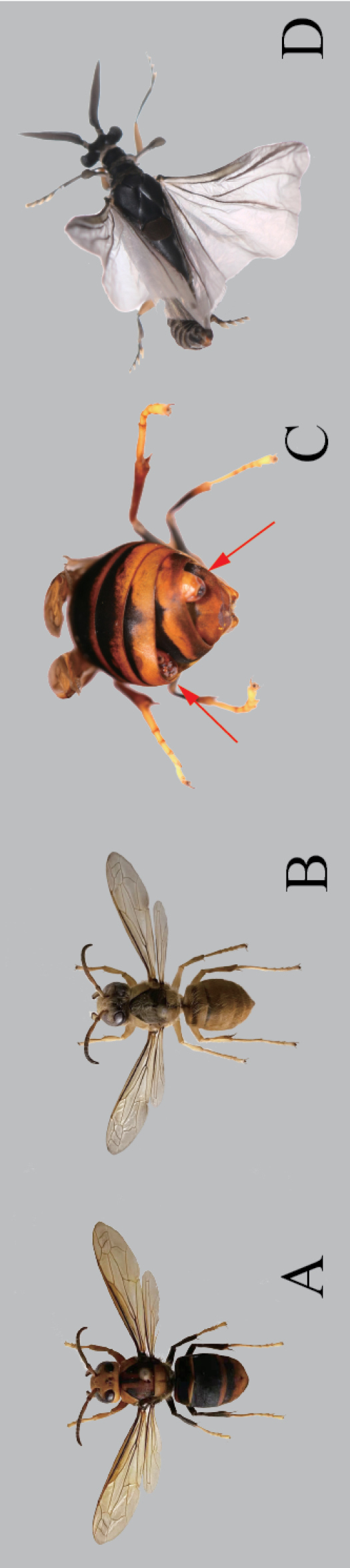
*Xenosyangi* Dong, Liu & Li sp. nov. and its host wasp. **A***Vespavelutina***B***Vespabicolor***C** wasp host parasitized by the new species (red arrows: male puparium (left), female(right)) **D** living male. (dorsal view).

In December 2019, some wasps (*Vespavelutina* Lepeletier, 1836 and *Vespabicolor* Fabricius, 1787) were collected by local villagers in southern Gaoligong Mountains (Yunnan, China). We checked these wasp individuals and found some of them parasitized by twisted-wing parasites. We collected male adults (Figs 1–3), cephalotheca of male puparium (Fig. 4), and neotenic females (Fig. 4) of these twisted-wing parasites from the abdomen and nests of their wasp hosts (Fig. 5). We assembled the mitogenome of a neotenic female from a *V.velutina* nest using Next-generation technologies, and found that the mitogenome sequence is similar to that of *X.vesparum* in our previous work (Zhang et al. 2021). In this study, we further make a close morphological examination of males and neotenic females and cephalotheca of male puparium, and further assembled mitogenome of a male from a *V.velutina* nest and another neotenic female from a *V.bicolor* nest to compare them with that of the neotenic female from a *V.velutina* nest (Zhang et al. 2021). Our morphological and molecular results revealed that these adults of different sexes and different hosts are associated with the same species of *Xenos*, and is new to science.

## ﻿Materials and methods

### ﻿Specimens

The male and neotenic female specimens of the new species *Xenosyangi* Dong, Liu & Li, sp. nov. were collected from the nests of both *V.velutina* and *V.bicolor* in Gaoligong Mountains, Xiangda Township, Longling County, Yunnan Province in December, 2019. The type materials of the new species described in this paper are deposited in the Insect Collection of Kunming Institute of Zoology, Chinese Academy of Sciences, Kunming, China (KIZ). Information on the other seven *Xenos* species (*X.oxyodontes*, *X.moutoni*, *X.vespularum*, *X.pecki*, *X.vesparum*, *X.ropalidiae*, *X.minor*) in the phylogenetic analysis was obtained from previous reports (Carapelli et al. 2006; McMahon et al. 2011; Nakase and Kato 2013; Jůzová et al. 2015; Benda et al. 2019, 2021). In detail, male and neotenic females of *X.oxyodontes* (*COI* GenBank accessions number: AB759562–AB759569; JN082805; MK431184; MN914546) were collected from Japan and Korea (McMahon et al. 2011; Nakase and Kato 2013; Benda et al. 2019, 2021); male pupa, males and neotenic females of *X.moutoni* (*COI* GenBank accessions number: AB759570–AB759582, MN914545, MK431183) were collected from China, Japan and Laos (Nakase and Kato 2013; Benda et al. 2019, 2021); two males of *X.vespularum* (*COI* GenBank accessions number: AB759583; MK431222) were collected from Japan (Nakase and Kato 2013; Benda et al. 2021); male and neotenic females of *X.pecki* (*COI* GenBank accessions number: MN914547–MN914549; MK431187) were collected from USA (Benda et al. 2019, 2021); male and neotenic females of *X.vesparum* (*COI* GenBank accessions number: DQ364229.1; KF803535.1; MN914557; JN082806; MN914561; MK431205) were collected from Italy, Czech Republic, Austria (Carapelli et al. 2006; Jůzová et al. 2015; Benda et al. 2019, 2021); two neotenic females of *X.ropalidiae* (*COI* GenBank accessions number: MK431185–MK431186) were collected from Laos and Nepal, and two males of *X.ropalidiae* (*COI* GenBank accessions number: MK431189-MK431190) were collected from Malaysia (Benda et al. 2019); and male and neotenic females of *X.minor* (*COI* GenBank accessions number: MN914559–MN914560; MN914569) were collected from Croatia (Benda et al. 2021).

### ﻿Morphological description

Images of the living adults were taken using a Canon 70D camera in conjunction with a Canon EF 100 mm f/2.8L IS USM. The habitus images were taken using a stereomicroscope Nikon, SMZ18 equipped with NIS-Elements (Nikon, Japan). Scanning electron microscopes (SEM) images were taken using TM4000 II (Hitachi, Japan). The specimens used for SEM were directly fixed in 70% ethanol, and then dried at the room temperature. Morphological terminology follows those of Kinzelbach (1971), Kifune and Maeta (1985), Kathirithamby and Hughes (2006) and Koeth et al. (2012).

### ﻿DNA extraction, library construction, sequencing, mitogenome assembling and sequence comparison

Total genomic DNA of one male collected from *V.velutina* nest and one neotenic female collected from *V.bicolor* nest was extracted using a TIANamp Genomic DNA Kit (TIANGEN, China) based on manual instruction. Library construction, sequencing, mitogenome assembly follows those in our previous work (Zhang et al. 2021), in which the mitogenome of one neotenic female collected from *V.velutina* was sequenced. We assembled the nearly complete mitogenomes of both male and neotenic female individuals, and compared them with that in our previous work (Zhang et al. 2021). Then the mitogenome sequences of the three individuals were compared in pairs using BLAST in NCBI website.

### ﻿Phylogenetic analyses

*COI* is an useful molecular marker for species identification in many insects, including twisted-wing parasites (Nakase and Kato 2013; Jůzová et al. 2015; Benda et al. 2021, 2019). Here, we used the *COI* sequences from the nearly complete mitogenomes of one male and one neotenic female of *Xenosyangi* sp. nov. and another *Xenos* neotenic female individual in our previous work (Zhang et al. 2021) for the association between neotenic female and male adults.

Combined with 45 *COI* sequences of *Xenos* published by others (Carapelli et al. 2006; McMahon et al. 2011; Nakase and Kato 2013; Jůzová et al. 2015; Benda et al. 2021), phylogenetic analyses were performed using maximum likelihood (ML), and maximum parsimony (MP) methods with four strepsipteran species *Stylopsater* Reichert, 1914, *Melittostylopshesperapium* Kinzelbach, 1971, *Halictoxenostumulorum* Perkins, 1918 and *Crawfordiawarnckei* Kinzelbach, 1970 (Stylopidae) (GenBank Accession: GAZM00000000.2, MK431155, KF803415, MK431154) as outgroups (Misof et al. 2014; Jůzová et al. 2015; Benda et al. 2019) . Briefly, *COI* sequences were first translated to amino acid sequences with the invertebrate mitochondrial genetic code, and then aligned by codons using the ClustalW algorithm in MEGA-X v10.1.8 (Sudhir et al. 2018). Next, MEGA-X was also used to find the best nucleotide substitution model (“GTR+I”) and to reconstruct phylogenetic trees with the default parameters and 1000 bootstrap iterations.

## ﻿Results

### ﻿Sequences and phylogenetic analyses

We assembled nearly complete mitogenomes of one male adult collected from a *V.velutina* nest (15324 bp) (GenBank accession number: OK329871) and one neotenic female collected from a *V.bicolor* nest (14670 bp) (GenBank accession number: OK32987). The mitogenomes of these two individuals in this study and one neotenic female in our previous work (Zhang et al. 2021) contain the same sequence except for the A+T-rich region and a gap between *trnaM* and *trnaI*, suggesting the nature of the same species for these three individuals with different sexes and different host. In this study, the mitogenome of male adult was annotated as 13 protein-coding genes (PCGs), 22 transfer RNA genes (tRNAs) and two ribosomal RNA genes (rRNAs) and an A+T-rich region, while only 36 mitogenomic genes (excl. *trnM*) were annotated in the incomplete mitogenome sequence of the neotenic female.

**Figure 6.**
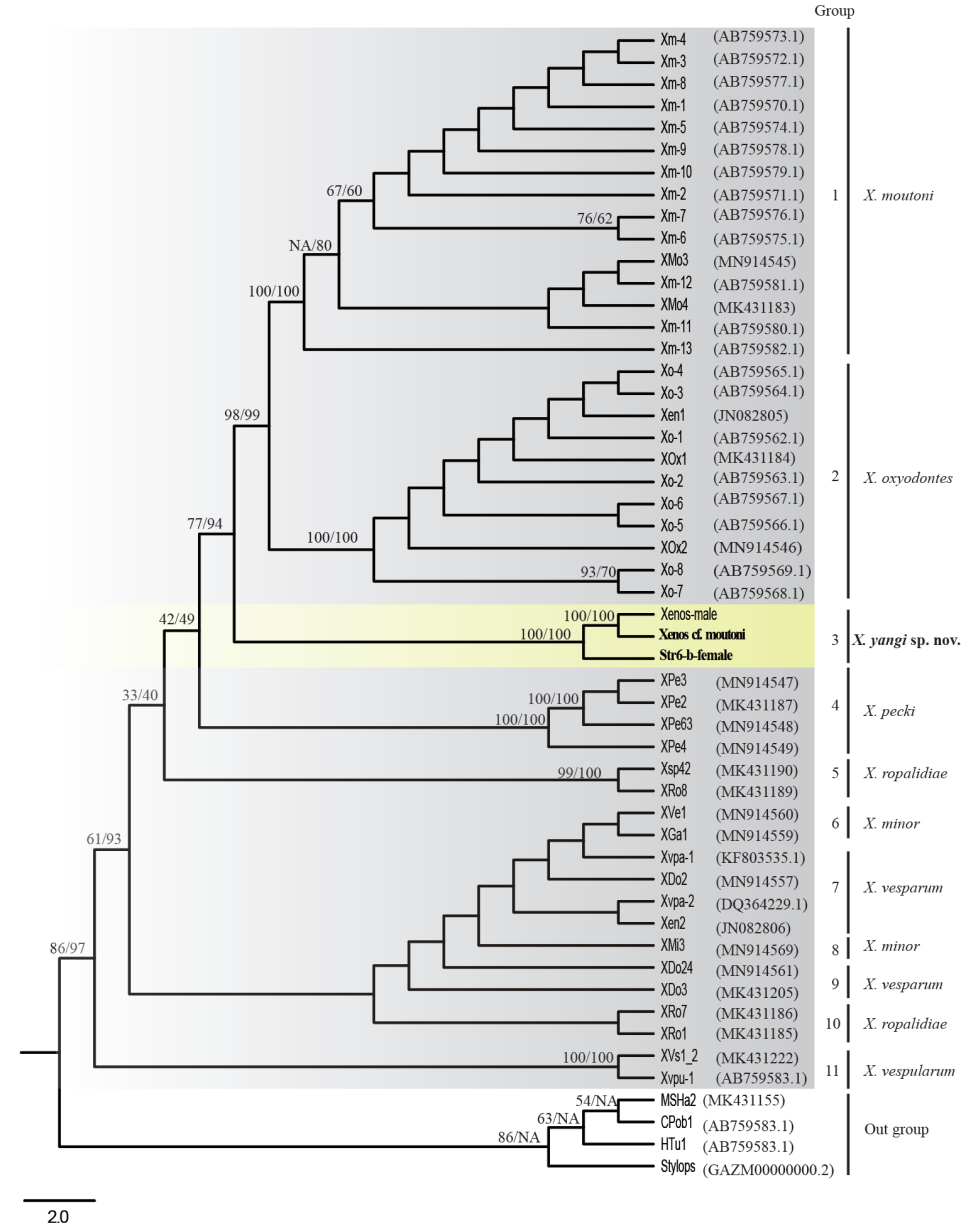
Phylogeny tree of *Xenos* species inferred from mitochondrial cytochrome *c* oxidase subunit 1 (*COI*) using Maximum parsimony method. In total, 48 *COI* sequences of different *Xenos* species were used to investigate their phylogenetic relationships. two sequences (str6-b-female and Xenos-male) were sequenced in this study, and that of *Xenoscf.moutoni* (MW222190.2) was sequenced in Zhang et al. (2021). Other 45 sequences were published by the following studies (Benda et al. 2021; McMahon et al. 2011; Nakase and Kato 2013; Jůzová et al. 2015; Carapelli et al. 2006). *Stylopsater* Reichert, 1914, *Melittostylopshesperapium* Kinzelbach, 1971, *Halictoxenostumulorum* Perkins, 1918 and *Crawfordiawarnckei* Kinzelbach, 1970 (outgroup) were used as outgroups. The phylogenetic trees were constructed using Maximum Parsimony (MP), and Maximum Likelihood (ML). Branch support values are described as Maximum Parsimony (MP)/Maximum Likelihood (ML) in MP tree.

We further extracted a major fragment (1518 bp) of *COI* sequences from three Chinese *Xenos* individuals (one male and two neotenic females), and combined 45 *COI* sequences of identified species of *Xenos* reported by others to make the dataset for the phylogenetic analyses using ML and MP methods. All phylogenetic trees show that the three Chinese *Xenos* individuals (one male individual from the *V.velutina* nest and two female individuals from the *V.velutina* and *V.bicolor* nests) cluster together with high bootstrap values (Fig. 6). The genetic divergence among three Chinese *Xenos* individuals varies from 0 to 0.014 (Table 2, Fig. 6), which is equal to that among *X.moutoni* individuals and less than that among *X.oxyodontes* individuals. Especially, one male and one neotenic female from the same host nests (*V.velutina*) showed no genetic divergence, suggesting their conspecific identity. These findings confirm that these male and neotenic female individuals collected from different host populations are the same species. This species can be differentiated from all the other Eurasian species of *Xenos* based on the genetic analyses and further morphological examination, and thus stands as a new species described below.

## ﻿Taxonomy

**Xenidae Saunders, 1872**

### *Xenos* Rossius, 1793

#### ﻿ Xenos yangi

Taxon classificationAnimaliaStrepsipteraXenidae

Dong, Liu & Li sp. nov.

http://zoobank.org/41C69672-2AD0-4E04-8C8B-F1F2352813A9

Chinese name 杨氏胡蜂𧎥 Figs 1 , 2 , 3 , 4 , 5

##### Type locality.

China, Yunnan, Longling County, Xiangda Township.

##### Type materials.

***Holotype***: male (KIZ0130767), “Gaoligong Mountains, Xiangda Township, Longling County, Yunnan Province, 24.4441083 N, 98.7239194 E, 1666 m, 20.XII.2019, local villagers leg.”, kept in 75% ethanol, [red label]. (KIZ). ***Paratypes***: four males (KIZ0130768–KIZ0130771), three neotenic females (KIZ0130772–KIZ0130774), same data as holotype (KIZ), kept in 75% ethanol, [yellow label].

##### Other material examined.

One neotenic female, “Gaoligong Mountains, Xiangda Township, Longling County, Yunnan Province, 20. XII. 2019, local villagers leg.”, partially used for extracting genomic DNA (accession number MW222190; Zhang et al. 2021). One neotenic female and one male, “Gaoligong Mountains, Xiangda Township, Longling County, Yunnan Province, 20. XII. 2019, local villagers leg.”, both partially used for extracting genomic DNA in this study.

##### Diagnosis.

**Male.** Head transverse. Antenna (Fig. 2B) four-segmented, 1^st^ with distal lateral extension and wider than 2^nd^, 3^rd^ and 4^th^ flabellate with subequal length. Palpus twice as long as maxilla (Fig. 2C). Mandible (Fig. 2D) slender, widened at base, tapering at tip. Prescutum pentagonal. Scutellum longitudinally elongated, triangular. Proventrite posteromedially with a small U-shaped notch, forming a pair of small lobes (Fig. 2H). Mesoventrite posteromedially bifurcated into a pair of long digitiform projections (Fig. 2I). Tarsus four-segmented, without claws (Fig. 2E–G). Penis colter-shaped (Fig. 2J). **Cephalotheca of male puparium** (Fig. 4A). Maxillae almost oval, bigger than mandible. Clypeus furrowed and close to mandible. Antenna half size of eye. **Neotenic female** (Fig. 4B–D). Cephalothorax almost rectangular, 3/4 strongly contracted; birth opening, protuberance (Fig. 4C); apex of mandibles straight (Fig. 4D).

##### Description.

**Male** (Fig. 1). ***Length*** 5.6 mm (holotype), 5.5–8.1 mm (paratypes) (combined length of head, pronotum and abdomen). ***Coloration*** (Fig. 1A, B): head, antenna, maxillary palpus, coxa, and abdomen black; femur, tibia and tarsus brown; hind wing semi-transparent. ***Head*** transverse, 1.44 mm in width. ***Compoundeye*** raspberries-like, each composed of about 84 ommatidia, ommatidiaprominent and separated by chitinous bridges covered with micortrichia (Fig. 3A). ***Antenna*** four-segmented (Fig. 2B), scapus wider than pedicellus, scapus with distal lateral extension, pedicellus half as long as scapus, 3^rd^ and 4^th^ flabellate with subequal length, hirsute (Fig. 3B). ***Mandible*** (Fig. 2J) smooth, sword-like, gradually thicker from middle until 3/4, and then sharply tapering at tip. ***Maxillaeandpalpus*** (Figs 2C, 3B) covered with short hairs, palpus twice half as long as maxillae, palpus narrower. ***Pronotum*** (Fig. 1A) quadrangular with a protuberant apex. ***Acrotergit*** (Fig. 1A) with two ends turned up, central depression. ***Mesonotum*** (Fig. 1A) saddle-shaped, central M-shaped depression, pseudo-haltere on both sides. ***Prescutum*** (Fig. 1A) pentagonal with round tops. ***Scutellum*** (Fig. 1A) acutely triangular. ***Postlumbium*** (Fig. 1A) broad, generally rounded, but emarginate anteriorly. ***Postnotum*** (Fig. 1A) triangular.

***Hind wing*** sector with nine veins (Fig. 2A). C and Sc fused, half length of costal margin. R1 and R2 veins almost glued together, R2 vein extending from middle to wing apex; R3 vein from middle to outer margin of wing; R4 vein terminated at distal 1/4 of the wing and approximating R5 vein. MA, CuA1, CuA2 and CuP veins present and uninterrupted.

***Proventrite*** laterally with anepisternum angulately curved at middle, and posteromedially with a small U-shaped notch, forming a pair of short lobes (Fig. 2H); ***Mesoventrite*** with basisternum transversely rectangular, anterolaterally roundly prominent, posterolaterally hook-like, sternellum broadly rhombic, posteriorly bifurcated into a pair of long digitiform projections (Fig. 2I). ***Foreleg*** (Fig. 2E) coxa expands, trochanterofemur with a protuberance near coxa, tibia longer than femur, widened near tarsus, tarsus four-segmented, 1^st^ tarsomere with oval pit outside, 4^th^ tarsomer without claws. ***Midleg*** (Fig. 2F) coxa as long as trochanterofemur, other parts similar to those of foreleg. ***Hind leg*** (Fig. 2G) trochanter half length of femur, femur strong. ***Abdomen*** 10-segmented as long as thorax, black; segment I tergites and sternites shrink; segment II–VIII sternites distinctly broader than tergites, segment IX narrower than segment VIII, with caudally elongated subgenital plate; segment X tube-like, curved. Anus flat. Penis colter-shaped (Fig. 2J).

**Cephalotheca of male puparium** (Fig. 4A). Cephalotheca elliptical. Maxilla almost oval, bigger than mandible. Clypeus furrowed and close to mandible. Antenna half size of eye.

**Neotenic female** (Fig. 4B–D). Length 11.0–16.0 mm, maximum breadth of abdomen about 4.5–5.0 mm (Fig. 4B); cephalothorax 2.2 mm in length and 1.76 mm in width (Fig. 4C, D). Coloration: cephalothorax brownish yellow, abdomen yellow. Cephalothorax almost rectangular, 3/4 strongly contracted; birth opening, protuberance (Fig. 4C); apex of mandible straight (Fig. 4D); abdomen slender, four birth organs.

##### Comparative notes.

Considering the geographic distance and host association of those species of Africa and Americas, we mainly compared the male adult, the cephalotheca of the male papurium, and the neotenic female of this new species with ten described known species distributed in Asia (nine species) and Europe (one species) (Table 1). These species were originally described based on the male adult, the cephalotheca of the male papurium, and/or the neotenic female (Table 1). The new species can be distinguished from *X.moutoni* (China: Yunnan, Anhui, Taiwan), *X.dianshuiwengi* (China: Fujian), *X.formosanus* (China: Taiwan), *X.provesparum* (Indonesia) and *X.oxyodontes* (Japan) based on the external characters of male adult. The male adult of *X.moutoni* maxilla as long as palpus and the postlumbium is straight anteriorly and posteriorly (Kifune and Maeta 1985). The male adult of *X.oxyodontes* (Japan) has the postlumbium rounded anteriorly and posteriorly (Nakase and Kato 2013). The proventrite is not concaved in *X.dianshuiwengi* (China: Fujian), *X.formosanus* (China: Taiwan) and *X.provesparum* (Indonesia) (Kifune and Maeta 1985; Kifune 1986; Yang 1999).

**Table 1.** Distribution and described stages of 11 *Xenos* species from Asia and Europe. Literature in which the species was originally described is highlighted in bold.

Species	Distribution	Male	Cephalotheca of male puparium	Neotenic female	Primary larvae
*Xenosyangi* Dong, Liu & Li sp. nov.	China: Yunnan	This study	This study	This study	NA
*Xenosmoutoni* (Buysson, 1903)	China: Yunnan, Anhui, Taiwan	Kifune & Maeta, 1985	Buysson, 1904	**Buysson, 1903**	NA
*Xenoscircularis* Kifune & Maeta 1985	China: Taiwan	NA	NA	**Kifune & Maeta, 1985**	NA
*Xenosyamaneorum* Kifune & Maeta, 1985	China: Taiwan	NA	**Kifune & Maeta, 1985**	**Kifune & Maeta, 1985**	NA
*Xenosformosanus* Kifune & Maeta, 1985	China: Taiwan	**Kifune & Maeta, 1985**	**Kifune & Maeta, 1985**	**Kifune & Maeta, 1985**	NA
*Xenosdianshuiwengi* Yang, 1999	China: Fujian	**Yang, 1999**	NA	NA	NA
*Xenosoxyodontes* Yuta & Makoto 2013	Japan	**Yuta & Makoto, 2013**	**Yuta & Makoto, 2013**	**Yuta & Makoto, 2013**	NA
*Xenosvespularum* Kifune & Maeta, 1975	Japan	**Kifune & Maeta, 1975**	**Kifune & Maeta, 1975**	**Kifune & Maeta, 1975**	NA
*Xenoshebraei* Kinzelbach, 1978	India	NA	**Kinzelbach, 1978**	**Kinzelbach, 1978**	NA
*Xenosprovesparum* Kifune,1986	Indonesia	**Kifune, 1986**	**Kifune, 1986**	**Kifune, 1986**	NA
*Xenosvesparum* Rossius, 1793	Europe; Northern Africa	**Rossius, 1793**	**Rossius, 1793**	**Rossius, 1793**	Pohl & Beutel, 2005

NA: Not availabl

The new species can be distinguished from *X.circularis* (China: Taiwan), *X.yamaneorum* (China: Taiwan), *X.vespularum* (Japan), *X.hebraei* (India) and *X.vesparum* (Europe; Northern Africa) by the female cephalothorax. It is almost circular or ovoid in *X.yamaneorum*, *X.circularis*, *X.vespularum* and *X.vesparum* (Kifune and Maeta 1975; Kifune and Maeta 1985). The female cephalothorax is slightly wider than long in *X.hebraei*. Besides that, this new species can be also separated from *X.yamaneorum* and *X.vespularum* by the oval maxillae of the male cephalotheca (the two compared species lack the oval maxillae of the male cephalotheca).

##### Distribution.

China (Yunnan).

##### Biology.

The hosts of this new species are *Vespavelutina* (Fig. 5A) and *Vespabicolor* (Fig. 5B). It parasitizes in the host abdomen. Its body partly protrudes from the portion between the two abdominal segments of the hosts. One wasp can usually carry 1–4 parasite individuals (Fig. 5C). After emergence, male adults fly away from their hosts (Fig. 5D). Neotenic females remain in the host’s abdomen with their anterior cephalothorax protruding. When neotenic females are removed from their host abdomen, they can be seen to be covered with larval exuviae.

##### Etymology.

The specific epithet is dedicated to the late famous Chinese entomologist Chi-Kun Yang, who made significant contributions to the studies on Strepsiptera in China.

## ﻿Discussion

**Figure 7.**
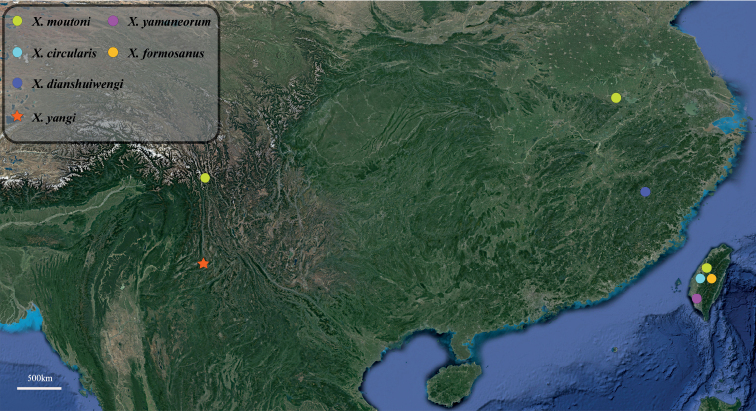
Distribution of the *Xenos* species from China.

Due to the discovery of *X.yangi* sp. nov., the number of Chinese *Xenos* species increases to six (Fig. 7) while the Asian species add up to ten. In general, the Asian *Xenos* species are endoparasites of Vespinae (yellow jackets and hornets) and Polistinae (paperwasps) (Suppl. material 1: Table S1). *Vespa* (Vespinae) and *Polistes* (Polistinae) are common hosts for most *Xenos* species (Cook 2019). Except two Taiwanese species parasiting on *Polistes*, all other eight Asian species parasite on *Vespa*. Considering the species diversity of Vespinae and Polistinae in China (Carpenter 2011), we confirm the rich *Xenos* species diversity in China.

Among the 10 Asian *Xenos* species, six species (including the new species here) are described based on both males and neotenic females, one species solely based on males, two species are based on neotenic females and the cepholotheca of the male puparium, and one species is solely based on neotenic females (Table 1). This situation in describing new species based only on neotenic females is also common in the taxonomy of *Xenos* from Africa and America (Suppl. material 1: Table S1). Considering the sexual dimorphism in twisted-wing parasites it is feasible to describe a new *Xenos* species when both male and female specimens are available. Thus, the association of both sexes and different stages of development in the same species of *Xenos* is crucial for future studies. This study provide an example of associating both sexes using combined biological, morphological and molecular evidence.

*Xenosmoutoni* was originally described by Buysson (1903) based on only neotenic female specimens collected in Anhui (Ngan-hoei = Anhui Prov., Yng-chan = Xuanchen?宣城) and Yunnan (Yun-nam = Yunnan Prov., Tsé-kou = Cigu茨古 (Xu and Qiu 2020). Then, Buysson (1904) recorded its male puparium cephalotheca based on the specimens collected from the type locality. Kifune (1985) redescribed the male adult and cephalotheca of the male puparium of this species from Taiwan. In Buysson’s work, the cephalotheca of the male puparium might be the main diagnostic trait to identify Taiwan *X.moutoni*. However, the author did not give a detailed description of the male puparium cephalotheca. According to available male specimens, cephalotheca of the male puparium and the neotenic females of the new species in the present study, we compared the different stages of the new species with the description of a male adult (Taiwan), cephalotheca of the male paparium (type locality), or the neotenic female (type locality) of *X.moutoni*, facilitating the delimitation of these two species both recorded from Yunnan. In addition, our study affirms again that molecular data, e.g., the DNA barcodes, are essential for the association of dimorphic sexes and different developmental stages in twisted-wing parasites taxonomy.

In the molecular data analysis, we noticed that different populations of five monophylic species (*X.moutoni*, *X.oxyodontes*, *X.yangi*, *X.pecki*, and *X.vespularum*) show genetic divergence of less than 0.036 (Table 2). Especially for *X.moutoni*, the genetic divergence among their different populations from Laos, China, Japan is less than 0.014 (Table 2). For the other lineages including specimens identified as *X.minor*, *X.vesparum* and *X.ropalidiae*, we noticed that *X.minor* and *X.vesparum* form a clade including four groups (group 6, 7, 8 and 9) (Fig. 6) and their genetic divergences are less than 0.007 (Table 2), suggesting these specimens may be the same species (Benda et al. 2021). On the other hand, different populations (Laos, Nepal, Malaysia) of *X.ropalidiae* form two separate groups (5 and 10) with a genetic divergence of 0.368–0.421 (Table 2), which may include different species (Benda et al. 2021). These findings suggest that an integrated methodology of molecular, biological, and morphological evidence should be adopted in taxonomy of such endoparasites as twisted-wing insects.

**Table 2.** Summary of pairwise distances based on *COI* sequences among different *Xenos* species.

Group	Species	1	2	3	4	5	6	7	8	9	10	11
1	*X.moutoni*	0–0.014	–	–	–	–	–	–	–	–	–	–
2	*X.oxyodontes*	0.111–0.123	0–0.071	–	–	–	–	–	–	–	–	–
3	*X.yangi* sp. nov.	0.191–0.208	0.269–0.287	0–0.014	–	–	–	–	–	–	–	–
4	*X.pecki*	0.322–0.344	0.369–0.382	0.338–0.361	0–0.036	–	–	–	–	–	–	–
5	*X.ropalidiae*	0.330–0.348	0.308–0.341	0.302–0.333	0.326–0.358	0.089	–	–	–	–	–	–
6	*X.minor*	0.425–0.432	0.451–0.465	0.497–0.508	0.544–0.560	0.367–0.377	0	–	–	–	–	–
7	*X.vesparum*	0.425–0.440	0.451–0.473	0.497–0.516	0.544–0.568	0.367–0.385	0–0.003	0–0.003	–	–	–	–
8	*X.minor*	0.425–0.432	0.451–0.465	0.497–0.508	0.544–0.560	0.367–0.377	0	0–0.003	NA	–	–	–
9	*X.vesparum*	0.411–0.434	0.439–0.475	0.494–0.507	0.526–0.588	0.358–0.393	0.001–0.003	0.001–0.007	0.001–0.003	0.005	–	–
10	*X.ropalidiae*	0.275–0.383	0.329–0.404	0.382–0.433	0.452–0.474	0.368–0.421	0.435–0.517	0.435–0.525	0.435–0.517	0.437–0.552	0.2	–
11	*X.vespularum*	0.481–0.505	0.451–0.478	0.4472–0.478	0.549–0.577	0.458–0.472	0.608–0.612	0.608–0.616	0.608–0.612	0.590–0.608	0.519–0.523	0.001

## Supplementary Material

XML Treatment for Xenos yangi

## Supplementary materials

Supplementary material 1 Table S1 Data type xlsx file Explanation note Known species of Xenos and their geographical distribution, type depository and host. https://binary.pensoft.net/file/641104This dataset is made available under the Open Database License (http://opendatacommons.org/licenses/odbl/1.0/). The Open Database License (ODbL) is a license agreement intended to allow users to freely share, modify, and use this Dataset while maintaining this same freedom for others, provided that the original source and author(s) are credited.Zhiwei Dong, Xingyue Liu,Chuyang Mao, Jinwu He, Xueyan Li

## References

[B1] Benda D Nakase Y Straka J (2019) Frozen antarctic path for dispersal initiated parallel host-parasite evolution on different continents. Molecular Phylogenetics and Evolution 135: 67–77. 10.1016/j.ympev.2019.02.023 30849429

[B2] Benda D Votýpková K Nakase Y Straka J (2021) Unexpected cryptic species diversity of parasites of the family Xenidae (Strepsiptera) with a constant diversification rate over time. Systematic Entomology 46: 252–265. 10.1111/syen.12460

[B3] Buysson R (1903) Note pour servir à l’histoire des Strepsiptères. Bulletin de la Société entomologique de France 8(9): 174–175. 10.3406/bsef.1903.23277

[B4] Buysson R (1904) Monographie des guêpes ou *Vespa*. Annales de la Societe Entomologique de France 73(3): 85–634.

[B5] Carapelli A Vannini L Nardi F Boore JL Beani L Dallai R Frati F (2006) The mitochondrial genome of the entomophagous endoparasite *Xenosvesparum* (Insecta: Strepsiptera). Gene 376(2): 248–259. 10.1016/j.gene.2006.04.005 16766140

[B6] Carpenter JM Dvořák L Kojima J Nguyen LTP Perrard A Pickett KM (2011) Taxonomic notes on the Vespinae of Yunnan (Hymenoptera: Vespidae). American Museum Novitates 3709: 1–10. 10.1206/3709.2

[B7] Cook JL (2014) Review of the biology of parasitic insects in the order Strepsiptera. Comparative Parasitology 81(2): 134–151. 10.1654/4723.1

[B8] Cook JL (2019) Annotated catalog of the order Strepsiptera of the world. Transactions of the American Entomological Society 145(2): 121–267. 10.3157/061.145.0202

[B9] Cook JL Mayorga-Ch D Sarmiento CE (2020) A new species of *Xenos* (Strepsiptera: Xenidae) from Colombia, with comments on the neotropical species of the genus. Transactions of the American Entomological Society 146(2): 331–336. 10.3157/061.146.0204

[B10] Jůzová K Nakase Y Straka J (2015) Host specialization and species diversity in the genus *Stylops* (Strepsiptera: Stylopidae), revealed by molecular phylogenetic analysis. Zoological Journal of the Linnean Society 174(2): 228–243. 10.1111/zoj.12233

[B11] Kathirithamby J (2018) Biodiversity of Strepsiptera. In: Foottit RG Adler PH (Eds) Insect biodiversity: science and society.Vol II. John Wiley & Sons, New York, 673–703. 10.1002/9781118945582.ch22

[B12] Kathirithamby J (2021) World Strepsiptera Database. https://strepsiptera.aphia.org [accessed on 2021-08-23]

[B13] Kathirithamby J Hughes DP (2006) Description and biological notes of the first species of *Xenos* (Strepsiptera: Stylopidae) parasitic in *Polistescarnifex* F. (Hymenoptera: Vespidae) in Mexico. Zootaxa 1104(1104): 35–45. 10.11646/zootaxa.1104.1.3

[B14] Kinzelbach RK (1971) Morphologische Befunde an Fächerflüglern und ihre phylogenetische Bedeutung (Insecta: Strepsiptera). Zoologica 41(119/126): 1–256.

[B15] Kinzelbach RK (1978) Insecta, Fächerflügler (Strepsiptera). Series: Die Tierwelt Deutschlands, vol. 65. G. Fischer Verlag, Jena, 166 pp.

[B16] Kifune T Maeta Y (1975) A New Subgenus and New Species of the Genus *Xenos* (Strepsiptera, Stylopidae) from Japan : Studies on the Japanese Strepsiptera II. Japanese Journal of Entomology 43(4): 446–455.

[B17] Kifune T Maeta Y (1985) Taxonomical studies on the genus *Xenos* (Strepsiptera, Stylopidae) parasitic on *Vespa* and *Polistes* (Hymenoptera, Vespidae) of Taiwan with descriptions of three new species: Notulae Strepsipterologicae XIV. Japanese Journal of Entomology 53: 426–435.

[B18] Kifune T (1986) A new species of the genus *Xenos* (Strepsiptera, Stylopidae) parasitic on the genus *Provespa* (Hymenoptera, Vespidae) from West Sumatra, Indonesia. Japanese Journal of Entomology 54: 84–88.

[B19] Koeth M Friedrich F Pohl H Beutel RG (2012) The thoracic skeleto-muscular system of *Mengenilla* (Strepsiptera: Mengenillidae) and its phylogenetic implications. Arthropod Structure & Development 41(4): 323–335. 10.1016/j.asd.2012.04.005 22583792

[B20] Manfredini F Giusti F Beani L Dallai R (2007) Developmental strategy of the endoparasite *Xenosvesparum* (Strepsiptera, Insecta): host invasion and elusion of its defense reactions. Journal of Morphology 268(7): 588–601. 10.1002/jmor.10540 17437299

[B21] McMahon DP Hayward A Kathirithamby J (2009) The mitochondrial genome of the ‘twisted-wing parasite’ *Mengenillaaustraliensis* (Insecta, Strepsiptera): a comparative study. BMC Genomics 10: e603. 10.1186/1471-2164-10-603 PMC2800125 20003419

[B22] McMahon DP Hayward A Kathirithamby J (2011) The first molecular phylogeny of Strepsiptera (Insecta) reveals an early burst of molecular evolution correlated with the transition to endoparasitism. PLoS ONE 6(6): e21206. 10.1371/journal.pone.0021206 PMC3125182 21738621

[B23] Misof B Liu S Meusemann K Peters RS Donath A Mayer C Frandsen PB Ware J Flouri T Beutel RG Niehuis O Petersen M Izquierdo-Carrasco F Wappler T Rust J Aberer AJ Aspock U Aspock H Bartel D Blanke A Berger S Bohm A Buckley TR Calcott B Chen J Friedrich F Fukui M Fujita M Greve C Grobe P Gu S Huang Y Jermiin LS Kawahara AY Krogmann L Kubiak M Lanfear R Letsch H Li Y Li Z Li J Lu H Machida R Mashimo Y Kapli P McKenna DD Meng G Nakagaki Y Navarrete-Heredia JL Ott M Ou Y Pass G Podsiadlowski L Pohl H von Reumont BM Schutte K Sekiya K Shimizu S Slipinski A Stamatakis A Song W Su X Szucsich NU Tan M Tan X Tang M Tang J Timelthaler G Tomizuka S Trautwein M Tong X Uchifune T Walzl MG Wiegmann BM Wilbrandt J Wipfler B Wong TKF Wu Q Wu G Xie Y Yang S Yang Q Yeates DK Yoshizawa K Zhang Q Zhang R Zhang W Zhang Y Zhao J Zhou C Zhou L Ziesmann T Zou S Li Y Xu X Zhang Y Yang H Wang J Kjer KM Zhou X (2014) Phylogenomics resolves the timing and pattern of insect evolution. Science 346: 763–767. 10.1126/science.1257570 25378627

[B24] Nakase Y Kato M (2013) Cryptic diversity and host specificity in giant *Xenos* strepsipterans parasitic in large *Vespa* hornets. Zoological Science 30(4): e331. 10.2108/zsj.30.331 23537244

[B25] Pohl H Beutel RG (2005) The phylogeny of Strepsiptera (Hexapoda). Cladistics 21(4): 328–374. 10.1111/j.1096-0031.2005.00074.x 34892965

[B26] Pohl H Beutel RG (2008) The evolution of Strepsiptera (Hexapoda). Zoology 111(4): 318–338. 10.1016/j.zool.2007.06.008 18356032

[B27] Richter A Beutel RG Pohl H (2017) The female cephalothorax of *Xenosvesparum* Rossi, 1793 (Strepsiptera: Xenidae). Arthropod Systematics and Phylogeny 75(2): 327–347.

[B28] Rossius P (1793) Observations de M. Rossi sur un nouveau genre d’insecte, voisin des ichneumons. Bulletin des sciences de la Société Philomathique de Paris 1: e49. [1 plate]

[B29] Sudhir K Glen S Li M Christina K Koichiro T (2018) Mega x: molecular evolutionary genetics analysis across computing platforms. Molecular Biology & Evolution 6: e6. 10.1093/molbev/msy096 PMC5967553 29722887

[B30] Xu H Qiu JY (2020) Three new synonyms within the flower chafer genus *Goliathopsis* Janson, 1881 (Coleoptera: Scarabaeidae: Cetoniinae) from China. Zootaxa 4789(1): 91–131. 10.11646/zootaxa.4789.1.3 33056445

[B31] Yang CK (1999) Strepsiptera. In: Huang BK (Ed.) Fauna of insect in Fujian porvince of China.Fujian science & technology press, Fujian. Vol. 3, 184–185. [In Chinese]

[B32] Zhang R Li J Mao CY Dong ZW He JW Liu GC Zhao RP Wang W Li XY (2021) The mitochondrial genome of one ‘twisted-wing parasite’ Xenoscf.moutoni (Insecta, Strepsiptera, Xenidae) from Gaoligong Mountains, Southwest of China. Mitochondrial DNA, Part B 6(2): 512–514. 10.1080/23802359.2021.1872443 33628908 PMC7889101

